# Association Between SARS-CoV-2–Related Experiences and Smoking Cessation in Switzerland: A Repeated Cross-Sectional Study

**DOI:** 10.3390/ijerph23020198

**Published:** 2026-02-03

**Authors:** Eloïse Cuvit, Margot Guth, Semira Gonseth Nusslé, Valérie D’Acremont, Carole Clair

**Affiliations:** 1 Department of Ambulatory Care, Center for Primary Care and Public Health (Unisanté), University of Lausanne, 1010 Lausanne, Switzerland; margot.guth@unisante.ch (M.G.); semira.gonseth@gmail.com (S.G.N.); carole.clair@unisante.ch (C.C.); 2 Global and Environmental Health Sector, Center for Primary Care and Public Health (Unisanté), University of Lausanne, 1010 Lausanne, Switzerland; valerie.dacremont@unisante.ch

**Keywords:** smoking cessation, COVID-19, Switzerland

## Abstract

**Highlights:**

**Public health relevance—How does this work relate to a public health issue?**

- Smoking remains a major global public health burden, causing over 8 million deaths worldwide each year.
- The COVID-19 pandemic has influenced various health-related behaviors, including smoking, creating a timely context for this study.

**Public health significance—Why is this work of significance to public health?**

- Understanding the determinants of smoking cessation is essential to leverage every opportunity to promote cessation.
- This is the only study to specifically explore changes in tobacco-related behaviors during the COVID-19 pandemic—from the first wave to the period prior to the launch of large-scale vaccination—in Switzerland.

**Public health implications—What are the key implications or messages for practitioners, policy makers and/or researchers in public health?**

- Individuals seeking SARS-CoV-2 testing may be more health-conscious, which could contribute to smoking cessation.
- Pandemics may represent critical windows of opportunity for smoking cessation interventions that should be actively leveraged by healthcare professionals, especially general practitioners, and by public health authorities, through clear and tailored messages.

**Abstract:**

The COVID 19 pandemic may have influenced smoking behaviours, including decisions to quit smoking. This study aimed to investigate smoking cessation following the first two waves of the COVID-19 pandemic in Switzerland and to assess whether cessation differed according to participants’ SARS-CoV-2–related experiences. Data from SérocoViD, a Swiss repeated cross-sectional study comprising five surveys in the canton of Vaud, was used. A total of 2454 participants aged 15 years and older from the first (May–July 2020) and third (February 2021) surveys were included. Association between SARS-CoV-2 infection experiences and cigarette smoking cessation were analyzed using logistic regression; both factors were unadjusted and adjusted for age and gender. Overall, 21.2% of participants reported being ex-smokers, but only a small proportion of the entire study population (i.e., including both smokers and non-smokers) reported quitting during the pandemic (0.5% in the first sample, 1.5% in the second). Participants who were smokers before the pandemic and had undergone diagnostic testing for SARS-CoV-2 showed a trend toward smoking cessation during the pandemic (non-adjusted odds ratio = 2.15; 95% confidence interval: 0.79–5.87). No such trends were found with a positive diagnostic test or serological result, or with COVID-19-like symptoms. These findings suggest that individuals seeking testing may be more health-conscious, potentially contributing to smoking cessation. For these individuals, the pandemic may represent a critical opportunity to promote smoking cessation, which should be leveraged by healthcare professionals and public health policies.

## 1. Introduction

On 11 March 2020, the World Health Organization (WHO) officially declared coronavirus disease-2019 (COVID-19)—caused by severe acute respiratory syndrome coronavirus 2 (SARS-CoV-2)—a pandemic [1]. Like many other countries, the Swiss government implemented a range of preventive public health measures and subsequently declared temporary lockdowns to limit viral transmission and protect vulnerable populations [2,3]. Since then, COVID-19 has remained a major public health concern. At the time of writing, the cumulative number of cases worldwide exceeds 776 million, with more than 7 million deaths, despite the availability of vaccines [4,5].

The tobacco epidemic represents another major global public health burden, responsible for over 8 million deaths worldwide annually [6]. Smoking is well-known to weaken pulmonary immune defenses, increasing individuals’ susceptibility to infections and the severity of outcomes for those infected [7]. Since the emergence of SARS-CoV-2, many studies have investigated the effect of smoking—both current and former—on the risk and severity of COVID-19. Evidence suggests that smoking is independently associated with an increased risk of mortality in patients with COVID-19 [8]. However, findings differ between current and former smokers. Former smokers are at increased risk of hospitalization, disease severity and mortality from COVID-19, while analyses regarding current smokers are inconclusive but suggest a positive association with disease progression, particularly among young adults [8,9,10,11]. In a meta-analysis by Patanavanich et al. [11], which included seven studies, distinguishing current from former smokers, there was a positive association between current smoking and COVID-19 progression.

Regarding the impact of COVID-19 pandemic on smoking behaviors, several studies focused on changes in tobacco consumption during the first COVID-19 lockdown in 2020. On one hand, many of these studies [12,13,14,15,16,17] report an increase in tobacco use among a portion of smokers, ranging from 18.5% to 45.8%, often as a coping strategy to manage the pandemic-related stress, anxiety and depression. On the other hand, some studies [18,19,20] report that some smokers reduced their consumption or attempted to quit smoking due to the pandemic. However, a systematic review by Bakaloudi et al. [21], showed that nearly half (i.e., 34/77) reported an increase in smoking habits during lockdown.

Beyond the first pandemic wave, few studies have assessed the evolution of smoking habits throughout the entire COVID-19 pandemic. Their findings [22,23,24], mainly based on sales data, self-reported use of nicotine replacement therapy (NRT), self-reported smoking cessation, or visits to smoking cessation clinics, remain inconsistent. Some studies [25,26], conducted in China at the end of 2020 and in England in spring 2021, highlight a decrease—rather than an increase—in the number of cigarettes smoked daily, and report that 4.5% and 17.5% of smokers, respectively, quit smoking.

To the best of our knowledge, no peer-reviewed studies have examined changes in smoking behavior—particularly smoking cessation—during the COVID-19 pandemic in Switzerland. This Swiss study addresses this gap by examining smoking cessation following the first and second waves of the pandemic. Specifically, it aims to investigate the association between smoking cessation reported after the first two waves and SARS-CoV-2–related experiences.

## 2. Materials and Methods

### 2.1. Study Design

This research is a secondary analysis of SérocoViD, a repeated cross-sectional observational study conducted in the canton of Vaud (a French-speaking region of Switzerland) during the COVID-19 pandemic (May 2020–July 2022). SérocoViD is part of Corona Immunitas, a nationwide program of SARS-CoV-2 seroprevalence and seroepidemiologic studies in Switzerland [27].

The SérocoViD study included five surveys with age-stratified participants, representative of the general population of the canton of Vaud. The first survey took place between 3 May and 7 July 2020, followed by the second from 20 October to 12 December 2020, and the third from 1 February to 6 February 2021. The fourth and fifth surveys occurred after the beginning of this research. The first and third surveys involved different cohorts of participants. In the second survey, only selected adult participants from the general population in the first survey were invited to participate (see Section 2.2. for details).

In January 2022, data were extracted from the first and third SérocoViD surveys. Data from the second survey were not used, as they provided only partial follow-up to the first survey, while data from the fourth and fifth surveys were not available at the time of analysis.

The SérocoViD study protocol was approved by the Ethics Committee of the canton of Vaud (CER-VD) (protocol number 2020-00887).

### 2.2. Study Population

Participants aged 15 years or older from the first and third SérocoViD surveys were included in this analysis. This age criterion was applied to ensure sample consistency, as only participants aged 15 and older were invited to participate in the third survey, whereas the first survey had no age restriction. Eligibility for the SérocoViD study required residence in the canton of Vaud.

The first survey included both the general population and specific subpopulations, defined as individuals living or working in conditions where social distancing was difficult to apply and who were at higher risk of exposure to COVID-19 (described below). In contrast, only the general population was included in the third survey. The general population sample was randomly drawn from the residential registry of the canton of Vaud, and was stratified by predefined age groups. Diplomats, individuals with a foreign address, asylum seekers, people with short-term residence permits, and elderly residents of nursing homes were excluded from the registry.

Specific subpopulations included confirmed COVID-19 cases and their household or close contacts, asylum seekers and employees in sectors such as food retail, public transportation, post office and laundry services. These subpopulations were sampled from 200 symptomatic COVID-19 patients with a positive reverse transcription polymerase chain reaction (RT-PCR) test, recorded in the cantonal registry during the first five weeks of the pandemic. This group included all the cases from the first 10 days, randomly selected cases from weeks 2, 3, 4, and 5, and all cases involving children, along with their close contacts identified through active tracing. Additionally, employees from the predefined sectors and asylum seekers from two centers of similar size and location—one with a high number of reported COVID-19 cases and one with fewer—were randomly selected [27].

Participants with missing or inconsistent data on sociodemographic characteristics, tobacco use, or SARS-CoV-2 infection status were excluded from this analysis (see Annexes).

**Figure 1:**
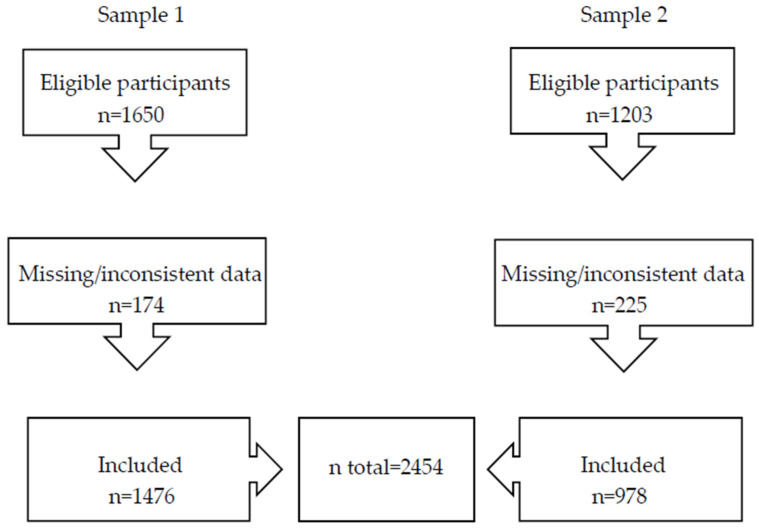
Flow diagram of participant inclusion.

In total, 2454 participants from the SérocoViD study were included in this analysis: 1476 from the first survey (Sample 1) and 978 from the third survey (Sample 2). A total of 174 and 225 participants were excluded from Samples 1 and 2, respectively, due to missing or inconsistent data. For the logistic regression analyses, the two samples were combined (Figure 1).

### 2.3. Variables

In the SérocoViD study, all participants were invited to complete a baseline self-administered questionnaire and undergo a SARS-CoV-2 serologic blood test, conducted either at study centers or at home [27]. The questionnaire collected detailed information on personal demographics and health status, including smoking habits, COVID-19-specific information (such as symptoms, hospitalization, medications and SARS-CoV-2 testing), socio-demographic characteristics and the economic impact of lockdown measures, on individuals in the immediate vicinity and their relevant symptoms, on preventive measures, as well as exposure and level of concern about the pandemic.

In the first survey, participants were asked about their current smoking status and frequency (daily, weekly or less), type of products used (cigarettes, electronic cigarettes (e-cigarettes) with or without nicotine, heated tobacco products, nicotine substitutes, and others), number of cigarettes smoked daily or the nicotine concentration in e-cigarettes (none, <6 mg/mL, 6–12 mg/mL, ≥13 mg/mL). Past smoking behavior were assessed, such as regular smoking for over six months, age at smoking initiation, and time since smoking cessation (within the past 12 months, 1–2 years ago, 2–5 years ago, 5–10 years ago, or more than 10 years ago). Additionally, participants reported how the lockdown affected their use of cigarettes or other nicotine products, including stopped use, reduced consumption, no change, increased consumption, resumed use, did not resume use, or never smoked/used such products.

In the third survey, questions focused on cigarette smoking status (daily smoker, occasional smoker, ex-smoker, never smoker, or having smoked fewer than 100 cigarettes in a lifetime), the number of cigarettes smoked per day or per month (for daily and occasional smokers, respectively), the date of smoking cessation for ex-smoker (month and year), current or past use of tobacco products other than cigarettes (daily, occasional, former or never), the types of products used, current e-cigarette use, and nicotine dosage in e-cigarettes (no nicotine, ≤6 mg/mL, >6 mg/mL but <13 mg/mL or ≥13 mg/mL).

For this analysis, we used the SARS-CoV-2 serologic tests results and self-reported data from the baseline questionnaire, which included sociodemographic characteristics, health status (comorbidities and smoking), episode(s) of symptoms consistent with SARS-CoV-2 infection, and history of diagnostic testing.

To assess whether cigarette smoking cessation occurred during the COVID-19 pandemic, we used either the self-reported cessation period and lockdown influence (from the first survey) or the reported cessation date (from the third survey) for each ex-smoker. The population quit rate was defined as the proportion of individuals who quit smoking among the entire study population, whereas the smoking cessation rate was defined as the proportion of quitters among smokers.

Sociodemographic variables—including gender, age, comorbidities, educational and professional status, household size, and use of e-cigarettes, heated tobacco, or other tobacco/nicotine products—were examined as potential confounder. For participants with missing data on comorbidities, former tobacco use, or household size, we assumed no comorbidities, no history of tobacco use and that they lived alone.

### 2.4. Statistical Analysis

Statistical analyses were performed using STATA/BE 17.0. Descriptive statistics were reported as means with standard deviation (SD) or medians with interquartile range (IQR) for continuous variables, and as percentages for categorical variables.

The association between SARS-CoV-2 infection—defined as the presence of COVID-19-compatible symptoms, a positive PCR test, or a positive serologic test—and cigarette smoking cessation (ex-smoker status) was assessed using logistic regression models and expressed as odds ratio (OR) and 95% confidence interval (CI).

Both univariate and multivariate logistic regression analyses were performed, with the latter adjusted for the effects of age and gender. To avoid unstable coefficient estimates, categories with fewer than five participants were merged when appropriate. To ensure robustness, logistic regression analyses restricted to smokers only (smoking cessation rate) were conducted. Statistical analyses were conducted using a significance of *p* < 0.05, with all *p*-values reported transparently. Given the limited sample size, findings were interpreted cautiously and described as “compelling trends” rather than definitive evidence, even if statistical significance was reached, due to the increased risk of Type I and Type II errors.

## 3. Results

### 3.1. Participant Characteristics

Baseline characteristics of participants in each sample are presented in Table 1. These characteristics are subsequently described and analyzed to compare the two samples, with a particular focus on smoking behavior and SARS-CoV-2–related experiences.

Gender distribution was similar across both samples (51.7% women in Sample 1 vs. 52.4% in Sample 2; *p* = 0.75). However, participants in Sample 2 were older on average (mean age—Sample 2: 50.2 years ± 21.75 vs. 47.2 years ± 17.69; *p* < 0.001). A higher proportion of participants in Sample 1 reported having no comorbidities (68.8% vs. 51.2%; *p* < 0.001).

With respect to professional status, a majority of participants in Sample 1 were employed or self-employed (61.7%), compared to 39.2% in Sample 2 (*p* < 0.001). Conversely, a larger proportion of Sample 2 participants had a university or higher education degree (EPFL/EPFZ or non-university higher education) (43.8% vs. 32.9% in Sample 1; *p* < 0.001). Household composition also differed: participants in Sample 1 were more likely to live with two or more people (53.5% vs. 43.8%) whereas those in Sample 2 were more likely to live with only one other person (40.1% vs. 28.5%) (*p* = 0.01).

Daily smoking rates were not significantly different between the two samples (12.9% in Sample 1 vs. 9.7% in Sample 2), nor was the proportion of ex-smokers (21.6% vs. 20.7%). During the COVID-19 pandemic, 7 participants (2 women, 5 men) in Sample 1 and 15 participants (10 women, 5 men) in Sample 2 reported having quit smoking. Although population quit rate during the pandemic was more frequent in Sample 2 (1.5% vs. 0.5% in Sample 1; *p* < 0.01), the overall proportion of individuals who quit smoking during this period, relative to all participants, remained low in both samples. The number of pre-pandemic smokers was estimated at 252 in Sample 1, including 191 daily smokers, 54 occasional smokers and 7 participants who reported quitting during the pandemic, and 161 in Sample 2, including 95 daily smokers, 1 occasional smoker and 15 who reported quitting during the pandemic. This rate was estimated under the assumption that few individuals initiated smoking during the pandemic, based on evidence from a previous meta-analysis [28]. Consequently, the smoking cessation rate was low, with 2.8% of pre-pandemic smokers (7 out of 252) in Sample 1 and 9.3% (15 out of 161) in Sample 2 reporting having quit smoking during the pandemic. Overall, 5.3% of smokers (22 out of 413) reported smoking cessation during the pandemic.

The use of alternative tobacco or nicotine products was low in both samples. E-cigarette use was uncommon (1.8% in Sample 1 vs. 2.1% in Sample 2; *p* = 0.50), as was the use of heated tobacco products (1.5% vs. 1.7%; *p* = 0.63) and other tobacco/nicotine-containing products (2.5% vs. 3.5%; *p* = 0.16).

Participants in Sample 2 were more likely to report at least one episode of symptoms compatible with SARS-CoV-2 infection (64.0% vs. 42.5% in Sample 1; *p* < 0.001) and to have undergone a diagnostic testing (40.8% vs. 21.9%; *p* < 0.001). However, among those tested, a greater proportion of Sample 1 participants received a positive result (55.9% vs. 25.1%; *p* < 0.001). Finally, seroprevalence was slightly higher in Sample 1 (28.9%) compared to Sample 2 (22.5%; *p* < 0.001).

### 3.2. Logistic Regression Analysis

Logistic regression analysis was conducted to assess the association between SARS-CoV-2 infection and smoking cessation rate. The results of the univariate logistic regression analysis are presented in Table 2.

A compelling trend was observed between the use of e-cigarette, heated tobacco, or other tobacco/nicotine-containing products and smoking cessation rate during COVID-19 pandemic (OR 3.99; CI: 1.59–10.00), although the wide confidence interval reflects substantial uncertainty regarding the precise magnitude of this association. A similar trend was observed among participants who had undergone a prior SARS-CoV-2 diagnostic test (OR = 2.15; 95% CI: 0.79–5.87). While this interval spans modest to relatively large associations, the estimate suggests a possible link between testing experience and smoking cessation.

Participants who reported at least one episode of symptoms compatible with SARS-CoV-2 infection or a positive result from a prior SARS-CoV-2 diagnostic test and/or from the serology performed during the SérocoViD study had ORs of 1.27 and 1.28, respectively, for smoking cessation during the pandemic, but the wide 95% CI included the null (95% CI: 0.54–3.01 and 0.46–3.59, respectively). Similarly, participants with comorbidities and those with higher education had ORs above 1 (OR = 2.03; 95% CI: 0.86–4.81, and OR = 1.66; 95% CI: 0.69–3.99, respectively) although again with wide 95% CI including 1. Finally, the OR for men versus women was 0.81 (95% CI: 0.34–1.92), with the interval covering both lower and higher odds, indicating high uncertainty.

The results from the logistic regression analysis adjusted for age and gender were consistent with those from the univariate analysis (see Table S1).

## 4. Discussion

Among 2454 respondents, only a small proportion (0.9%) of the whole population of including smokers and non-smokers reported having quit smoking during the COVID-19 pandemic. The smoking cessation rate tended to be higher among smokers who had previously undergone a SARS-CoV-2 diagnostic test or who used alternative tobacco or nicotine products. However, no such trends were found between cessation and either experiencing COVID-19-like symptoms or testing positive on a previous diagnostic test and/or serology during the survey.

Overall, smoking cessation remained limited, with 5.3% of smokers reporting having quit smoking during the pandemic. Other studies have reported varying cessation rates: a large cross-sectional study conducted in China by Yang et al. [25] found a similar cessation rate of 4.5% in October 2020, whereas a longitudinal study conducted in England by Kale et al. [26] reported a higher cessation rate of 17.5% at 12-month follow-up (May–June 2021).

According to data from the Swiss Federal Statistical Office [29], smoking prevalence in the Canton of Vaud decreased from 28.2% in 2017 to 23.2% in 2022, representing a total reduction of 5 percentage points over five years. These figures are derived from repeated cross-sectional surveys and do not reflect individual-level changes over time but rather provide snapshots of population-level prevalence at different time points. While direct comparisons with our findings are limited by methodological differences, the observed decline in smoking prevalence is broadly consistent with our data, which showed that a small proportion of smokers had quit by early 2021, during the pandemic period.

Our analysis showed a trend between having undergone a prior SARS-CoV-2 test and smoking cessation rate during the pandemic. However, no such trend was observed with a positive test result—either diagnostic or serological—or with experiencing COVID-19-like symptoms. These findings are consistent with those of Kale et al. [26], who reported no significant relationship between confirmed or suspected SARS-CoV-2 infection, or a high perceived risk of COVID-19, and smoking cessation. Yang et al. [25] found that over 90% of 533 individuals who quit or reduced smoking during the pandemic did so due to COVID-19-related health concerns. This supports the hypothesis that undergoing testing may reflect greater health awareness, which, rather than the test result or symptoms themselves, could have motivated smoking cessation. It is also possible that our findings represent only the “tip of the iceberg” and that a larger sample of quitters would reveal stronger associations between COVID-19-related experiences and smoking cessation during the pandemic. Future research should recruit larger samples of smokers to better assess this association. However, pandemic conditions were exceptional and do not reflect everyday circumstances. Future research on this topic will therefore likely need to rely on secondary analyses of existing COVID-19 cohorts or other opportunistic study designs in which comparable social and environmental constraints are present. Finally, adopting an intersectional approach with larger samples of smokers may help to better understand disparities in smoking cessation across population subgroups and clarify how social positions and cumulative vulnerabilities shaped quitting behaviors.

We also found a compelling trend between the use of e-cigarettes, heated tobacco products, or other alternative tobacco/nicotine products and smoking cessation rate. To our knowledge, this relationship has not been specifically examined in the context of the COVID-19 pandemic. However, findings from a Cochrane meta-analysis [30], which included 319 randomized controlled trials, lend support to our interpretation. This analysis reported high-certainty evidence that nicotine-containing e-cigarettes were associated with higher quit rates compared to control conditions. It also reported that nicotine patches and fast-acting NRT were more effective than control interventions. These findings may help explain why participants in our study who used such products tended to be more likely to quit smoking.

No trend was found between having one or more comorbidities and smoking cessation rate during the COVID-19 pandemic in our study. This contrasts with findings by Kale et al. [26], who reported that individuals without health problems were more likely to quit smoking at 12-month follow-up—though this association was only observed in unadjusted analyses. Their adjusted models did not confirm the association, nor did they find a link with quit attempt. Similarly, a U.S.-based study [22] found that individuals with two or more comorbidities were less likely to attempt quitting in 2020 compared to the previous year.

Consistent with our findings, other studies have reported no significant association between higher educational attainment and smoking cessation [26] or changes in smoking behavior [25] during the pandemic. Another study [18] also reported that the intention to quit smoking due to COVID-19 was not significantly linked with educational level. However, these findings should be interpreted with caution. Data from the Swiss Federal Statistical Office [31], collected between 1992 and 2022, clearly indicate a substantial decline in smoking rates among individuals with tertiary education. These results suggest that, in the specific context of the pandemic, usual social gradients in smoking cessation may have been attenuated.

With regard to gender, in our study, the OR for men versus women was 0.81 with a large 95% CI indicating high uncertainty about gender difference in quitting smoking during the pandemic. This is in line with Kale et al. [26], who found no significant gender difference in smoking cessation. Conversely, Yang et al. [25] reported that male smokers were significantly less likely to reduce their smoking during the pandemic than female smokers. In terms of age, our findings reflected differences in cessation behavior during the pandemic, in line with those of Kale et al. [26]. However, no trends were found between smoking cessation rate and either employment status or household size in our study. These results are consistent with those of Guignard et al. [15], who also found no association between these sociodemographic factors and changes in smoking behavior during the COVID-19 lockdown in France.

This study has several limitations. First, the number of participants who reported quitting smoking during the COVID-19 pandemic was low, which likely reflects the secondary nature of this analysis, based on the SérocoViD study. Importantly, the study was designed to be representative of the general population of the Canton of Vaud and was not specifically focused on smokers. As a result, overall population quit rate (calculated across both smokers and non-smokers) underestimates the true smoking cessation statistic among smokers. To avoid diluted associations, we conducted logistic regression analyses restricted to participants who were smokers prior to the pandemic (smoking cessation rate). These analyses further reduce the sample size, limiting statistical power and increasing potential Type I/II errors. For this reason, observed associations should be interpreted as exploratory rather than conclusive, and are described as compelling or exploratory trends throughout our study. Nevertheless, these findings may still provide meaningful insights for future research and hypothesis generation. Reporting both population-level and smoker-specific analyses offers a complementary perspective on smoking behaviour changes during the pandemic. Second, the repeated cross-sectional design and the use of two distinct, non-overlapping samples preclude any longitudinal assessment of individual changes over time. These methodological features limit the interpretation of the temporality and direction of the observed associations; therefore, these results should be interpreted with caution. Additionally, all data—except for SARS-CoV-2 serology—were self-reported and may be subject to information bias. In particular, social desirability may have led to underreporting of tobacco use or differential misclassification of smoking cessation status, potentially biasing the estimated associations. However, given the low reported population quit rate and smoking cessation rate in our study, such bias is unlikely. Finally, this study did not collect detailed information on post-cessation use of other tobacco or nicotine products, which prevents assessment of whether participants who reported quitting smoking achieved complete nicotine abstinence or substituted with other products. Additionally, no data were collected on the rate of smoking cessation consultations with general practitioners, which could have influenced overall cessation outcomes. These remains an important area for future research. Other areas that were not addressed in this study include comparisons across specific subgroups to better understand which individuals were more likely to stop smoking. Such research could help identify populations that may require additional support, or conversely, identify factors associated with successful smoking cessation and clarify which forms of support are useful and effective during a pandemic or another event with comparable impacts.

Despite these limitations, the study has important strengths. To our knowledge, it is the only study to date that specifically explores changes in tobacco-related behaviors—particularly smoking cessation—during the COVID-19 pandemic using Swiss data. It is also one of the few to include data collected beyond the first wave of the pandemic. Most previous studies focused exclusively on the lockdown period and tended to report overall changes in smoking behavior without distinguishing between cessation, decreased, or increased consumption. Another strength is the wide range of sociodemographic factors considered. Furthermore, the study provides detailed information on types of tobacco and nicotine use, including frequency and product type, whereas most prior research only distinguished between traditional cigarettes and e-cigarettes. This study also took place prior to the launch of the large-scale vaccination campaign in Switzerland and offers insights into behavioral responses adopted during a prolonged period of pandemic-related stress.

## 5. Conclusions

Our findings suggest a trend between having undergone a SARS-CoV-2 diagnostic test and smoking cessation rate during the COVID-19 pandemic. This may indicate that certain smokers are more likely to quit depending on the health context or their health-related behaviours. Individuals who engage in health-seeking behaviours may be more responsive to cessation messages during periods of heightened health awareness. Consequently, such periods could be considered as potential windows of opportunity for smoking cessation interventions. Healthcare professionals, particularly general practitioners and public health authorities, could leverage these moments to deliver clear and targeted cessation support.

## Figures and Tables

**Table 1:** Baseline characteristics of participants.

Baseline Characteristics	Sample 1 n = 1476	Sample 2 n = 978	*p* Value
Gender, n (%)			0.75
Woman	763 (51.7)	512 (52.4)	
Man	713 (48.3)	466 (47.6)	
Age, [y] mean (SD)	47.2 (17.69)	50.2 (21.75)	<0.001
Comorbidity, n (%)			<0.001
No comorbidity	1015 (68.8)	501 (51.2)	
One or more comorbidities	461 (31.2)	477 (48.8)	
High blood pressure	217 (14.7)	163 (16.7)	0.188
Diabetes	60 (4.1)	41 (4.2)	0.877
Cardiovascular diseases	71 (4.81)	69 (7.1)	0.019
Chronic respiratory diseases	54 (3.7)	75 (7.7)	<0.001
Immune system weakness	49 (3.3)	42 (4.3)	0.211
Cancer	15 (1.0)	27 (2.8)	0.001
Impaired renal function	12 (0.8)	1 (0.1)	0.018
Other diseases	175 (11.9)	303 (31.0)	<0.001
Pregnancy	8 (0.5)	3 (0.3)	0.393
Professional situation, n (%)			<0.001
Employed or independent worker	911 (61.7)	383 (39.2)	
In training/studying mainly, retired, unemployed or other	565 (38.3)	595 (60.8)	
Highest education attainment, n (%)			<0.001
No diploma, mandatory school (Certificate of end of secondary school) or secondary education *	991 (67.1)	550 (56.2)	
University, EPFL/EPFZ, non-university higher education (HES, HEP, etc.)	485 (32.9)	428 (43.8)	
People in the same household, n (%)			0.012
None or non-available	266 (18.0)	158 (16.2)	
One	421 (28.5)	392 (40.1)	
Two or more	789 (53.5)	428 (43.8)	
Cigarettes smoking, n (%)			0.07
Daily	191 (12.9)	95 (9.7)	
Occasional	54 (3.7)	51 (5.2)	
Ex-smoker	319 (21.6)	202 (20.7)	
Never or less than 100 cigarettes in my life	912 (61.8)	630 (64.4)	
Population quit rate during COVID-19 pandemic, n (%)	7 (0.5)	15 (1.5)	0.006
Smoking cessation rate during COVID-19 pandemic, n (%)	n = 252 7 (2.8)	n = 161 15 (9.3)	0.004
Electronic cigarettes use, n (%)	26 (1.8)	21 (2.1)	0.495
Heated tobacco use, n (%)	22 (1.5)	17 (1.7)	0.631
Other tobacco/nicotinic products use, n (%)	37 (2.5)	34 (3.5)	0.161
Episode(s) with SARS-CoV-2 infection-compatible symptoms, n (%)	628 (42.5)	626 (64.0)	<0.001
Prior SARS-CoV-2 diagnostic test, n (%)	n = 424	n = 978	<0.001
No/Do not know/Do not want to answer this question	331 (78.1)	579 (59.2)	
Yes	93 (21.9)	399 (40.8)	
Result of prior SARS-CoV-2 diagnostic test, n (%)	n = 93	n = 399	<0.001
Negative/Do not know/Do not want to answer this question	41 (44.1)	299 (74.9)	
Positive	52 (55.9)	100 (25.1)	
Result of serologies, n (%)			<0.001
Negative/Undetermined	1050 (71.1)	758 (77.5)	
Positive	426 (28.9)	220 (22.5)	

SD = standard deviation, y = years old, EPFL/EPFZ = Swiss Federal Institute of Technology in Lausanne/Zurich, HES = universities of applied sciences, HEP = teacher training colleges, SARS-CoV-2 = severe acute respiratory syndrome coronavirus 2, * Secondary education = Professional training, Diploma, Maturity, Baccalaureate, International baccalaureate, professional maturity or equivalent.

**Table 2:** Univariate logistic regression analysis of smoking cessation rate, pre-pandemic smokers of SérocoViD, n = 413.

	Smoking Cessation Rate During COVID-19 Pandemic n = 22 (5.3%) ^1^			
Variables	n (%)	Odds Ratio	95% CI	*p* Value
Sample				
Sample 1	7 (31.8)	Reference		
Sample 2	15 (68.2)	3.6	1.43–9.03	0.006
Gender				
Woman	12 (54.5)	Reference		
Man	10 (45.5)	0.81	0.34–1.92	0.637
Age	22 (100)	0.97	0.95–1.00	0.061
Comorbidity				
No comorbidity	11 (50)	Reference		
One or more comorbidities	11 (50)	2.03	0.86–4.81	0.107
Professional situation				
Employed or independent worker	15 (68.2)	Reference		
In training/studying mainly, retired, unemployed or other	7 (31.8)	0.64	0.25–1.60	0.340
Highest education attainment				
No diploma, mandatory school (Certificate of end of secondary school) or secondary education ^2^	13 (59.1)	Reference		
University, EPFL/EPFZ, non-university higher education (HES, HEP, etc.)	9 (40.9)	1.66	0.69–3.99	0.257
People in the same household				
None or non-available	5 (22.7)	Reference		
One	5 (22.7)	0.86	0.24–3.06	0.816
Two or more	12 (54.6)	1.15	0.39–3.36	0.798
Electronic cigarettes, heated tobacco or other tobacco/nicotinic products use				
No	14 (63.6)	Reference		
Yes	8 (36.4)	3.99	1.59–10.00	0.003
Episode(s) with SARS-CoV-2 infection-compatible symptoms				
None	10 (45.5)	Reference		
One or more	12 (54.5)	1.27	0.54–3.01	0.588
Prior SARS-CoV-2 diagnostic test	n = 17			
No/Do not know/Do not want to answer this question	7 (41.2)	Reference		
Yes	10 (58.8)	2.15	0.79–5.87	0.135
Result of serologies and prior SARS-CoV-2 diagnostic test				
Negative/Undetermined/Do not know/Do not want to answer this question/Not available	17 (77.3)	Reference		
Positive (one of the two or both)	5 (22.7)	1.28	0.46–3.59	0.637

CI = confidence interval, EPFL/EPFZ = Swiss Federal Institute of Technology in Lausanne/Zurich, HES = universities of applied sciences, HEP = teacher training colleges, SARS-CoV-2 = severe acute respiratory syndrome coronavirus 2, ^1^ The two samples were combined, ^2^ Secondary education = Professional training, Diploma, Maturity, Baccalaureate, International baccalaureate, professional maturity or equivalent.

## Data Availability

Data is available upon request.

## Supplementary Materials

The following supporting information can be downloaded at https://www.mdpi.com/article/10.3390/ijerph23020198/s1, Table S1: Multivariate logistic regression analysis of smoking cessation rate.
