# Supplementary material for: Association Between SARS-CoV-2–Related Experiences and Smoking Cessation in Switzerland: A Repeated Cross-Sectional Study
Source: Int J Environ Res Public Health. 2026 Feb 3;23(2):198. doi: 10.3390/ijerph23020198 (PMC12940577; doi:10.3390/ijerph23020198)
Supplement: Supplementary file 1 [file ijerph-23-00198-s001.zip › Supplementary material - Table S1. Multivariate logistic regression analysis of smoking cessation rate.pdf]

Table S1. Multivariate logistic regression analysis of smoking cessation rate, pre-pandemic smokers of SérocoViD n = 413.

	Smoking cessation rate during COVID-19 pandemic adjusted by gender and age n = 22 (5.3%)[1]			
**Variables**	**n (%)**	**Odds Ratio**	**95% CI**	***p* Value**
Episode(s) with SARS-CoV-2 infection-compatible symptoms				
None	10 (45.5)		Reference	
One or more	12 (54.5)	1.12	0.47-2.70	0.798
Prior SARS-CoV-2 diagnostic test	n = 17			
No / Do not know / Do not want to answer this question	7 (41.2)		Reference	
Yes	10 (58.8)	1.91	0.69-5.33	0.215
Result of serologies and prior SARS-CoV-2 diagnostic test				
Negative / Undetermined / Do not know / Do not want to answer this question / Non-available	17 (77.3)		Reference	
Positive (one of the two or both)	5 (22.7)	1.2	0.43-3.38	0.732

CI=confidence interval, SARS-CoV-2=severe acute respiratory syndrome coronavirus 2, [1]The two samples were combined.
